# Testing for intersectional measurement invariance with the alignment method: Evaluation of the 8‐item patient health questionnaire

**DOI:** 10.1111/1475-6773.14189

**Published:** 2023-06-08

**Authors:** Dakota W. Cintron, Ellicott C. Matthay, D. Betsy McCoach

**Affiliations:** ^1^ Center for Integrative Developmental Science Cornell University Ithaca New York USA; ^2^ Department of Psychology Cornell University Ithaca New York USA; ^3^ Center for Opioid Epidemiology and Policy, Division of Epidemiology, Department of Population Health New York University School of Medicine New York New York USA; ^4^ Department of Educational Psychology University of Connecticut Storrs Connecticut USA

**Keywords:** evaluation design and research, gender/sex differences in health and health care, health equity, psychometrics, racial/ethnic differences in health and health care

## Abstract

**Objective:**

To demonstrate the use of the alignment method to evaluate whether surveys function similarly (i.e., have evidence of measurement invariance) across culturally diverse intersectional groups. Intersectionality theory recognizes the interconnected nature of social categories such as race, gender, ethnicity, and socioeconomic status.

**Data Sources:**

A total of 30,215 American adult's responses to the eight‐item Patient Health Questionnaire depression assessment scale (PHQ‐8) from the 2019 National Health Interview Survey (NHIS).

**Study Design:**

Using the alignment method, we examined the measurement invariance (equivalence) of the PHQ‐8 depression assessment scale across 16 intersectional subgroups defined at the intersection of age (under 52, 52 and older), gender (male, female), race (Black, non‐Black), and education (no bachelor's degree, bachelor's degree).

**Principal Findings:**

Overall, 24% of the factor loadings and 5% of the item intercepts showed evidence of differential functioning across one or more of the intersectional groups. These levels fall beneath the benchmark of 25% suggested for determining measurement invariance with the alignment method.

**Conclusions:**

The results of the alignment study suggest that the PHQ‐8 functions similarly across the intersectional groups examined, despite some evidence of different factor loadings and item intercepts in some groups (i.e., noninvariance). By examining measurement invariance through an intersectional lens, researchers can investigate how a person's multiple identities and social positions possibly contribute to their response behavior on an assessment scale.

**What is known on this topic**

- Intersectionality is a theoretical and analytic framework for understanding how multiple social and political factors (e.g., gender or race) intersect to affect an individual's lived experience.
- Measurement invariance means that a scale functions similarly across groups, contexts, or measurement occasions.
- No prior research has evaluated the measurement invariance of the widely used eight‐item Patient Health Questionnaire depression scale (PHQ‐8) across intersectional groups.

**What this study adds**

- Our research provides an empirical example of using the multiple‐group factor analysis alignment method to evaluate the intersectional measurement invariance of a scale.
- Because less than 25% of the factor loadings and intercepts were noninvariant, considered a benchmark for evaluating measurement invariance with the alignment method, the PHQ‐8 appears to function similarly across culturally diverse groups defined at the intersection of age, education, gender, and race.

1

Researchers often compare scale means across groups. However, comparing scale means is only appropriate if the scale measures the same construct (i.e., factor) in the same way across groups. Measurement invariance (or measurement equivalence) is the notion that an assessment scale (e.g., to measure depression) functions similarly across groups, contexts, or measurement occasions.^1^, ^2^, ^3^ To examine measurement invariance, researchers compare the hypothesized measurement model of the scale across two or more groups (or occasions) using confirmatory factor analysis. The measurement model describes the theorized relationship between the items of a scale and the construct or constructs the scale intends to measure.

Measurement invariance across groups/occasions is important for several reasons. Measurement invariance of a scale may provide evidence for the construct validity of a scale.^4^, ^5^ Evidence of measurement invariance can also help researchers determine whether group differences on a scale are meaningful or not. For example, one might wonder whether a difference on a scale across groups is real, or if it is related to differences in how the scale was measured in each group (e.g., measurement bias^6^). Critically, ignoring measurement noninvariance may result in invalid inferences and conclusions about the nature or magnitude of group differences on the construct of interest.^7^, ^8^

Intersectionality is a theoretical and analytic framework for understanding how multiple social identities (e.g., gender, race, and sexuality) intersect and reflect “social structures of oppression and privilege, such as sexism, racism, and heteronormativity.”^9^ Kimberlé Crenshaw coined the term in 1989 when advocating for the necessity of an intersectional legal framework for examining how race and gender interact to shape Black women's employment experiences and exposure to discrimination and violence.^10^, ^11^ Diverse fields such as epidemiology, psychology, and sociology are increasingly recognizing the importance of an intersectional approach to studying health and social inequalities.^12^, ^13^, ^14^, ^15^, ^16^, ^17^, ^18^, ^19^, ^20^

Recent recommendations suggest that *intersectionality* may be an important praxis for evaluating measurement invariance.^12^, ^13^, ^21^ Intersectional evaluations, however, pose important methodological challenges. The evaluation of measurement invariance typically involves one demographic variable (e.g., gender) with a few subgroups (male, female) using either multiple‐group confirmatory factor analysis (CFA) or Item Response Theory (IRT). Intersectional analysis, however, typically involves comparisons across many subgroups.^12^ For example, the intersection of race (White, Hispanic, Black, and Asian), gender (male, female), education (high school, bachelor's degree, graduate), and economic advantage (disadvantaged, advantaged) would result in 4 × 2 × 3 × 2 = 48 intersectional subgroups.

Researchers have noted that testing measurement invariance over many groups using multiple‐group CFA is “methodologically challenging.”^22^ The primary issue is how to handle multiplicity in comparing many groups. The most obvious issue is that the number of pairwise comparisons across measurement parameters (e.g., factor loadings, item intercepts) increases rapidly as the number of groups increases. Consequently, the chances of falsely concluding that there are between‐group differences in the measurement model increase. Also, research suggests that typical model fit criteria for evaluating the different levels of measurement invariance (e.g., Δ comparative fit index ≤0.01) may not be appropriate when the numbers of groups are large; these levels may be too stringent.^22^, ^23^ Asparouhov and Muthén^24^ also found that traditional multiple‐group CFA models with many groups led to many large modification indexes, which implies that a “long sequence of model modifications is needed to reach a model with acceptable fit and the search for a good model could easily lead to the wrong model.”

To overcome some of the challenges of handling many groups in a traditional multiple‐group CFA framework, Asparouhov and Muthén^24^, ^25^, ^26^ developed the alignment method. The alignment method optimizes the estimation of group‐specific factor means and variances without the requirement of exact measurement invariance (i.e., the alignment method allows for some degree of noninvariance). Consequently, the optimization approach of the alignment method eliminates several model‐building steps necessary in the traditional multiple‐group CFA approach to measurement invariance testing with many groups (e.g., model identification strategies across multiple models, the selection of anchor items, and the interpretation of many model comparisons).^8^ For these reasons, the alignment method seems well‐suited for evaluating measurement invariance of a scale at the intersection of multiple identities (e.g., gender, education, and race), as intersectional approaches tend to produce many distinct subgroups. (Note, the focus of the alignment method is on evaluating whether factor means and variance comparisons are meaningful across groups. If a researcher is interested in understanding whether a particular item is non‐invariant in some way, then the alignment approach may not be the most appropriate method, and a differential item functioning analysis may be more suitable.^18^)

## 2 MEASUREMENT INVARIANCE TESTING USING A TRADITIONAL MULTIPLE‐GROUP CFA APPROACH

The traditional multiple‐group CFA approach to measurement invariance testing involves fitting a series of nested measurement models. Confirmatory factor analysis produces several different types of parameters for a given measurement model. In this study, we focus on two types of item parameters: factor loadings and item intercepts. The *factor loading* is the direct effect of the factor on the item. In a single‐factor model, this is equivalent to the correlation between the factor and the item. The *item intercept* is the expected item mean for someone who is at the mean on the factor. Using a series of models that impose increasingly strict parameter constraints on the measurement model, we can evaluate the equality of measurement parameters across groups. The three levels of measurement invariance that are traditionally evaluated include configural, metric, and scalar.^8^, ^21^, ^27^

Configural invariance implies that the factor structure is the same for all groups (i.e., there are the same number of latent factors across groups, and the factors are measured by the same set of items in each group). Configural noninvariance indicates that either different constructs are being measured in different groups or the constructs of interest are measured by different sets of items across groups. In either case, comparisons of scale scores are not possible across groups.^8^ If we can establish configural invariance, we next evaluate metric invariance. Metric invariance constrains the factor loadings to be equal across groups. Metric invariance indicates that the strength of the relationship between latent factors and items is the same across the groups. If an item has a larger factor loading in one group, the factor better predicts item responses in one group than another. Evidence of metric noninvariance may lead to biases in observed factor variances, factor covariances, and factor means that can lead to incorrect conclusions in subsequent statistical inference.^8^, ^28^ Finally, scalar invariance imposes the same factor structure, factor loadings, and item intercepts of the item responses across groups. Scalar noninvariance implies that individuals from different groups who are equal on the latent trait differ in terms of their item responses. Comparisons of observed scale scores (e.g., mean or total scores) and factor scores assume scalar invariance.^8^, ^24^

## 3 MEASUREMENT INVARIANCE TESTING USING THE ALIGNMENT METHOD

The alignment method provides an alternative to the standard multiple‐group CFA approach for evaluating measurement invariance. The alignment method can estimate group‐specific factor means and variances without the requirement of exact measurement invariance. The emphasis on factor means and variances stems from researchers' desire to make unbiased comparisons of factor means and factor variances.^29^, ^30^, ^31^ The idea of the alignment optimization procedure is that an “adequate configural model”^8^ that has minimal differences in factor loadings and intercepts across groups should be sufficient for making group mean comparisons.^8^

To accomplish this, the alignment method identifies a configural model with minimal non‐invariance (i.e., a model where the factor loadings and item intercepts are as equivalent as possible across groups) that still allows for reliable (i.e., unbiased) factor mean comparisons across groups. In other words, the alignment method aims to minimize measurement noninvariance.^8^, ^24^ Traditional measurement invariance testing in a multiple‐group CFA framework requires a series of formal model comparisons (i.e., configural, metric, and scalar). In contrast, the alignment method uses the configural model and provides an *optimization* approach to measurement invariance evaluation.^8^, ^24^ After optimization, information about the invariance of every model parameter (i.e., the factor loadings and item intercepts) for every group is available. Based on Monte Carlo simulation studies, evidence suggests that fewer than 25% of parameters indicating noninvariance produces trustworthy results (i.e., one can make reliable factor mean and variance comparisons across groups).^25^, ^32^ The alignment method accordingly allows for the comparison of factor means and variances across groups while also allowing for minor measurement differences (approximate measurement invariance).^8^

Although the notion of evaluating intersectional measurement invariance has been recommended,^21^ the conceptual (e.g., subgroup choice) and statistical challenges inherent in evaluating measurement invariance with many groups (e.g., sample size and multiple group comparisons) may be barriers to implementation. Given the advantages of the alignment method for evaluating invariance with many groups, and building on the recommendations of Han et al.,^21^ we demonstrate the evaluation of *intersectional measurement invariance* using the alignment method. We illustrate the alignment method for intersectional measurement invariance testing with an empirical example using data on the eight‐item Patient Health Questionnaire depression assessment scale (PHQ‐8) depression scale from the 2019 National Health Interview Survey (NHIS).

## 4 METHODS

### 4.1 Participants

The National Health Interview Survey (NHIS) is one of the primary sources of information on the health and well‐being of American adults. The National Center for Health Statistics (NCHS) collects sample data from the civilian noninstitutionalized population of the United States. For illustrative purposes, we consider the age (≥52 or <52; 52 is the midpoint of the age range in NHIS), gender (male vs. female), race (Black vs. Non‐Black), and education (≥Bachelor's degree or <Bachelor's degree) of 2019 NHIS respondents as potential intersecting factors for evaluating intersectional measurement invariance. In total, there were 16 subgroups or intersections formed from these four variables (see Table 1). Note, we tried to include further granularity on race. For example, we considered an additional indicator of whether the participant was Hispanic or not. However, we found that the Non‐Hispanic Black male intersections had very small group sizes. Therefore, in this intersectional analysis, we focus primarily on Black versus Non‐Black comparisons.

**TABLE 1:** Intersectional group definitions and internal consistencies on PHQ‐8.

Group code	Group description	*N*	Internal consistency
1	<52 Black females with college degree	464	0.84
2	<52 Black females with no college degree	639	0.87
3	<52 Black males with college degree	294	0.86
4	<52 Black males with no college degree	472	0.86
5	<52 Non‐Black females with college degree	3654	0.84
6	<52 Non‐Black females with no college degree	2679	0.88
7	<52 Non‐Black males with college degree	3119	0.84
8	<52 Non‐Black males with no college degree	2817	0.87
9	52+ Black females with college degree	380	0.84
10	52+ Black females with no college degree	595	0.84
11	52+ Black males with college degree	498	0.84
12	52+ Black males with no college degree	209	0.87
13	52+ Non‐Black females with college degree	3717	0.84
14	52+ Non‐Black females with no college degree	4195	0.84
15	52+ Non‐Black males with college degree	3199	0.83
16	52+ Non‐Black males with no college degree	3284	0.84
	Overall	30,215	0.85

*Note*: Internal consistencies measured using Cronbach's alpha.

### 4.2 Measures

The PHQ‐8 depression scale is an established self‐report measure for assessing the severity of depressive disorders.^25^ Using Likert scaled items, the PHQ‐8 asks respondents how often, over the past 2 weeks, they were bothered by a set of indicators of depression (1 = not at all, 2 = several days, 3 = more than half the days, 4 = nearly every day). The item stems for the PHQ‐8 are in Table A1 and include indicators of depression such as “Little interest or pleasure in doing things” and “Feeling down, depressed, or hopeless.” Across all individuals within the current study, internal consistency reliability (i.e., Cronbach's alpha) was adequate (alpha = 0.85; see Table 1). Furthermore, within the intersectional subgroups, specific internal consistency reliability estimates were adequate (alpha range = 0.83–0.88; see Table 1).

### 4.3 Alignment method

We used Mplus version 8 to implement the alignment optimization procedure.^33^ This procedure uses two models (M0 and M1). M0 is the starting model and M1 is the optimized model. “M0 is produced by transforming a baseline configural model which assumes the same configuration of items to factors across groups.”^8^ Then the optimization procedure of the alignment method iteratively works to produce M1, where the differences between the factor loadings and item intercepts are minimized across groups. To evaluate the extent of noninvariance after the alignment optimization procedure has commenced, we can ascertain the extent of group differences in the factor loadings and intercepts. The approach used by Mplus is an “ad‐hoc” approach. Flake and McCoach^32^ provide a succinct account of this ad‐hoc approach:

After the group‐specific measurement models are estimated, invariance testing is conducted on all of the parameters. Taking one parameter at a time, two groups' parameter estimates are compared. If these estimates are not statistically significantly different from one another, they become connected. These comparisons are made again and again, across the groups' parameter estimates to create an invariant set, and then each parameter is tested against the mean of the invariant set. If, for that group, that parameter is statistically significantly different from the mean, then it is flagged as a noninvariant parameter. Asparouhov and Muthén (2014) controlled Type I error rate in the algorithm by setting the criterion value [alpha] to .001. For each parameter in the model, the output contains information about which groups are invariant, the mean differences between every pair of groups in the analysis, and the corresponding *p* value for the pairwise differences.

After the commencement of the ad‐hoc approach, several group differences in either the factor loadings or item intercepts would be indicative of a lack of measurement invariance. Asparouhov and Muthén^24^ found that up to 25% of noninvariance in either the factor loadings or item intercepts may result in “trustworthy” alignment results (i.e., reliable factor mean and variance comparisons across groups). Asparouhov and Muthén's findings were supported by Flake and McCoach,^32^ who found good performance when less than 29% of items are noninvariant. Thus, we use the 25% benchmark in our evaluation of the PHQ‐8.

The ad‐hoc approach in Mplus described above also produces an $R^{2}$ metric that is referred to as the invariance index. The invariance index indicates the degree of invariance of a given parameter and describes how far an individual parameter is from scalar invariance.^26^ Asparouhov and Muthén^24^ describe this as the degree to which “the variation across groups in the configural model intercepts and loadings for this item is explained by variation in the factor means and factor variance across groups.” An $R^{2}$ close to 1 for a parameter provides evidence that scalar invariance holds for that parameter^26^ because factor mean and variance differences across groups completely explain the between‐group variability in item parameters. Conversely, an $R^{2}$ near 0 provides evidence that the factor mean and variance differences across groups explain little to no between‐group variability in item parameters.^32^

**FIGURE A1:**
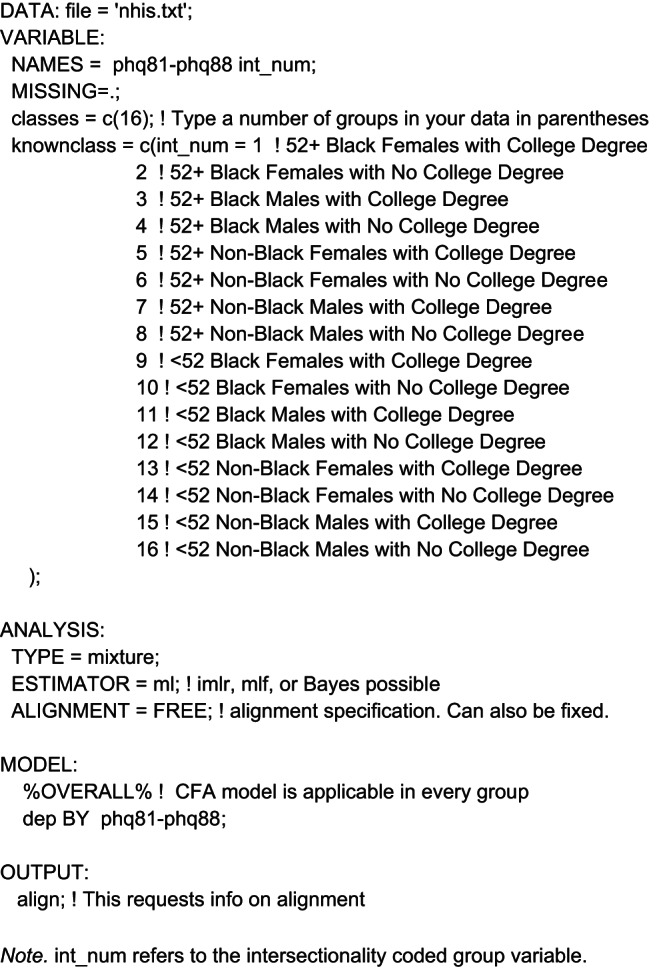
Mplus code for alignment method measurement invariance testing with continuous items.

Our code is available in Figure A1. In Mplus, there are two options to identify the alignment optimization procedure: FIXED and FREE. In the FIXED procedure, the factor mean and variance of the first group is fixed to either 0 or 1. In the FREE procedure, the factor mean of the first group is freely estimated. In this paper, we used the FREE procedure. because it has several advantages compared to the fixed alignment: (1) the FREE alignment method will always be more invariant than the fixed alignment, (2) the FREE alignment is independent of the reference group (i.e., changing the reference group does not alter the optimization method), and (3) Mplus will notify the user of whether the FREE procedure results are not trustworthy and that the FIXED procedure should be used instead.^24^, ^34^ Note, in all analyses, we treated items as continuous rather than categorical or ordinal (i.e., we use maximum likelihood estimation).^35^ In addition, the alignment method assumes an adequate configural model. We tested the configural model across all 16 intersectional groups and found it adequate.

## 5 RESULTS

Table 2 presents the results of the alignment analysis for the 16 intersectional groups and notes which item intercepts and loadings are non‐invariant in which groups. The results indicate that even after alignment, many item parameters remain noninvariant in several of the groups. Overall, we can see that 5% (6 out of 128) of the item intercepts are noninvariant across one or more groups and 24% (31 out of 128) of the factor loadings are noninvariant across one or more groups. Using the 25% benchmark,^24^ the results imply trustworthy alignment results for the PHQ‐8, meaning that group‐specific factor means and variances should be comparable across the 16 groups.

**TABLE 2:** Invariance results for aligned intercept and loading parameters for PHQ‐1 to PHQ‐8.

Item intercepts			
Item	Group invariance (non‐invariance)	# Non‐invariant groups	Invariance index
PHQ‐1	1 2 3 4 5 6 7 8 9 10 11 12 13 14 15 16	0	0.750
PHQ‐2	1 2 3 4 5 6 7 8 9 10 11 12 13 14 15 16	0	0.848
PHQ‐3	1 2 3 4 5 6 7 8 9 10 11 12 **(13) (14) (15)** 16	3	0.662
PHQ‐4	1 2 3 4 **(5) (6)** 7 8 9 10 11 12 13 **(14)** 15 16	3	0.670
PHQ‐5	1 2 3 4 5 6 7 8 9 10 11 12 13 14 15 16	0	0.818
PHQ‐6	1 2 3 4 5 6 7 8 9 10 11 12 13 14 15 16	0	0.608
PHQ‐7	1 2 3 4 5 6 7 8 9 10 11 12 13 14 15 16	0	0.841
PHQ‐8	1 2 3 4 5 6 7 8 9 10 11 12 13 14 15 16	0	0.636

Factor loadings			
Item	Group (non)‐invariance	# Non‐invariant groups	Invariance index
PHQ‐1	**(1) (2)** 3 4 **(5) (6)** 7 **(8)** 9 10 11 12 13 14 15 16	5	0.501
PHQ‐2	**(1) (2)** 3 4 **(5) (6)** 7 8 9 10 11 12 13 14 15 16	4	0.594
PHQ‐3	1 2 3 4 **(5)** 6 7 8 9 10 11 12 13 **(14)** 15 16	2	0.809
PHQ‐4	1 2 3 4 5 **(6)** 7 8 9 10 11 12 **(13)** 14 15 16	2	0.681
PHQ‐5	1 2 3 **(4)** 5 6 **(7) (8)** 9 10 11 12 13 **(14) (15) (16)**	6	0.632
PHQ‐6	1 2 3 4 **(5) (6) (7) (8)** 9 **(10)** 11 12 13 14 15 **(16)**	6	0.590
PHQ‐7	1 2 3 4 5 6 7 8 9 10 11 12 **(13)** 14 15 16	1	0.884
PHQ‐8	**(1)** 2 3 4 **(5)** 6 7 8 9 10 11 12 **(13) (14)** 15 **(16)**	5	0.341

*Note*: The group values correspond to the intersectional coding (see Table 1). The bolded numbers in parentheses refer to the groups that show significant non‐invariance for the parameter. The invariance index is *R* ^*2*^. An *R* ^*2*^. close to 1 provides evidence that there is complete invariance. Conversely, an *R* ^*2*^. near 0 provides evidence that group mean differences explain little to no variability in item parameters.

Table 2 reports the invariance index ($R^{2}$), which indicates the degree to which the between‐group variance in the factor means and factor variances explains the between‐group variance in the item intercepts and factor loadings. The invariance index ranged from 0.61 to 0.85 for the item intercepts and 0.34 to 0.88 for the factor loadings. The results indicate considerable invariance for the item intercepts: for many items, most of the between‐group variability in item parameters is explained by factor mean and variance differences across groups. However, there is a greater degree of noninvariance for the factor loadings relative to the item intercepts. Only the intercepts for items PHQ‐3 and PHQ‐4 showed evidence of noninvariance. However, factor loadings on every item displayed some noninvariance in at least one of the 16 groups.

Overall, regardless of education, non‐Black females were more likely to exhibit measurement noninvariance, and that invariance was typically for the factor loadings (i.e., the strength of the correlation between the factor and the items was considerably different for non‐Black females than for the remaining groups). The groups with the most noninvariance across item intercepts and factor loadings included younger non‐Black females with college degrees (group 5 had noninvariance on 1 item intercept and 5 factor loadings), younger non‐Black females with no college degree (group 6 had noninvariance on 1 item intercept and 4 factor loadings), older non‐Black females with a college degree (group 13 had noninvariance on 1 item intercept and 3 factor loadings), and older non‐Black females with no college degree (group 14 had noninvariance on 2 item intercepts and 3 factor loadings). Substantively, noninvariance in the item intercepts indicates that for non‐invariant groups, respondents with the same levels of overall depression have different means on the item. Likewise, non‐invariance in factor loadings means that for the noninvariant groups, the items either do a better or worse job discriminating between individuals in terms of their level of depression. However, the exact interpretation of these differences for item intercepts and factor loadings depends on the PHQ‐8 item and groups being compared.

**FIGURE 1:**
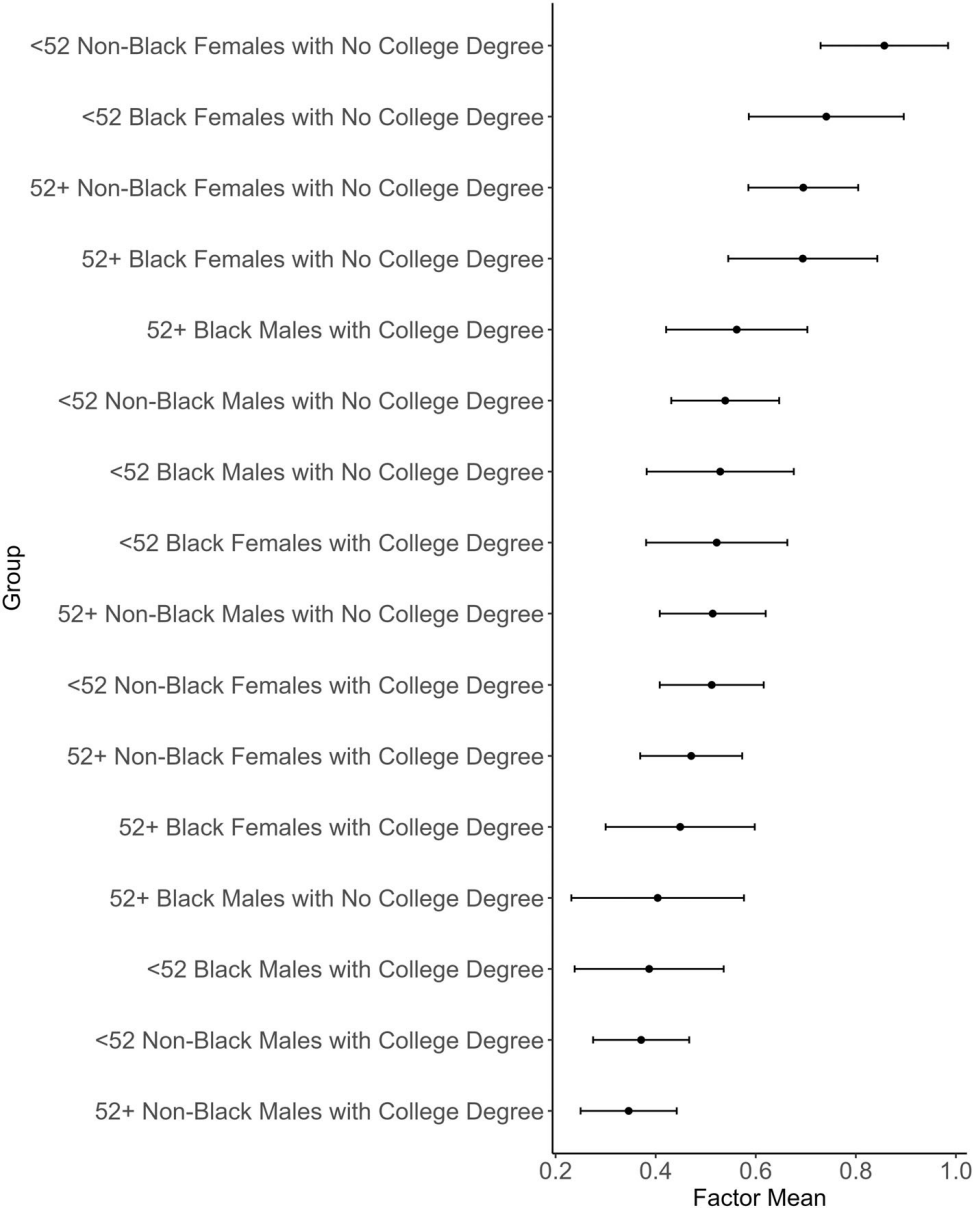
Plot of factor means by intersectional group. The factor means are for the construct of depression that is measured by the PHQ‐8 items. Smaller factor means are indicative of lower depression scores.

Lastly, the results of the alignment analysis also include a set of factor mean comparisons at the 5% significance level. (Factor means represent the intersectional subgroups' average levels of depression and are computed as a linear combination of the model parameters and the observed scores.) The intersectional subgroups' depression factor means are reported in Table 3 (and visualized in Figure 1). Comparing the 16 intersectional groups' depression factor means, we find that younger non‐Black females without a college degree in the study sample had higher depression scores on average than nearly all other groups; only younger Black females with no college degree had similar depression scores (see Table 3 and Figure 1).

**TABLE 3:** Intersectional group differences in factor means.

Ranking	Group code	Factor mean (SE)	Groups with significantly smaller factor mean
1	6	0.857 (0.065)	14 10 11 8 4 1 16 5 13 9 12 3 7 15
2	2	0.741 (0.079)	11 8 4 1 16 5 13 9 12 3 7 15
3	14	0.695 (0.056)	11 8 4 1 16 5 13 9 12 3 7 15
4	10	0.694 (0.076)	8 4 1 16 5 13 9 12 3 7 15
5	11	0.562 (0.072)	3 7 15
6	8	0.539 (0.055)	13 3 7 15
7	4	0.529 (0.075)	7 15
8	1	0.522 (0.072)	7 15
9	16	0.514 (0.054)	3 7 15
10	5	0.512 (0.053)	3 7 15
11	13	0.471 (0.052)	7 15
12	9	0.449 (0.076)	
13	12	0.404 (0.088)	
14	3	0.387 (0.076)	
15	7	0.371 (0.049)	
16	15	0.346 (0.049)	

*Note*: See Table 1 for group codes and descriptions. Factor mean comparisons are made at the 5% significance level in descending order. The factor means are for the construct of depression that is measured by the PHQ‐8 items. Smaller factor means are indicative of lower depression scores.

## 6 DISCUSSION

Intersectionality has been promoted as essential for advancing health disparities research. Bowleg noted five ways that intersectionality advances health disparities research: (1) it provides a linguistic and conceptual framework for understanding how multiple social identities intersect to shape health, (2) it motivates investigators to consider how complex social inequalities exist in the most marginalized groups, (3) it demonstrates how multiple identities intersect with macro‐level structural factors (e.g., poverty, racism, and sexism) to produce disparities in health outcomes, (4) it informs population‐level interventions and social policies to address the health needs of historically marginalized groups, and (5) it encourages data collection on health that can inform the analysis of intersecting social identities that facilitates more nuanced analyses of health disparities.^36^, ^37^

The notion of measurement invariance testing was introduced into the literature almost a century ago.^38^, ^39^, ^40^ In the development of assessment scales, measurement invariance testing has burgeoned into a critical step in ensuring that the scores on an assessment scale are not biased and their use in practice is justified. However, the use of measurement invariance testing has traditionally been limited to the evaluation of invariance across one demographic variable (e.g., gender) with few subgroups (male and female). This measurement invariance testing approach limits the advancement of our knowledge about a construct because it is not potentially sensitive to diverse groups' response behaviors.

In this paper, we argue for a framework of measurement invariance testing that is sensitive to the diverse identities of individuals by considering their identities at the intersection of several social and political identities (i.e., age, race, gender, and education). Using an intersectional framework for measurement invariance testing intentionally prompts investigators to thoughtfully consider how culturally diverse identities might interact with the main construct of the scale under evaluation. Our research demonstrates that using the alignment method for evaluating intersectional measurement invariance is feasible, and we highlight its implementation through an empirical analysis of the PHQ‐8.

As our empirical analysis demonstrates, the alignment method can provide evidence of the intersectional measurement invariance of a depression scale that is respectful of an individuals' multiple social identities. The results of our empirical intersectional alignment example provide evidence of the intersectional measurement invariance of the PHQ‐8, suggesting that the PHQ‐8 is likely appropriate for use across groups at the intersections of age, education, gender, and race. However, there was some evidence of noninvariance on some PHQ‐8 items for non‐Black females, which does indicate the need to consider why the PHQ‐8 may not be functioning similarly for this group.

Our research demonstrates how investigators might consider how individuals' intersecting social identities might contribute to differences in their responses to research scales. Consequently, the results of our study are important for advancing health disparities research by improving the sensitivity of psychometric procedures to identifying bias that may exist at the intersection of culturally diverse identities. Intersectional measurement invariance testing is critical to advancing health disparities research because it helps ensure valid evaluations of critical health and psychological constructs (e.g., depression) for individuals from diverse backgrounds and lived experiences. Further empirical analyses of the intersectional measurement invariance of assessment scales (e.g., anxiety or self‐esteem) is necessary.

### 6.1 Limitations

We used limited categorizations of age, race, gender, and education (i.e., each factor consisted of only two categories). Our choice of these limited identities and categorizations was largely due to sample size limitations. As the number of groups increases, so does the likelihood of small sample sizes in any given group, limiting the types of intersectional subgroups that can be evaluated. However, even though we found evidence of measurement invariance at the intersections of age, race, gender, and education for the PHQ‐8, there may be other identities not considered herein that could result in large degrees of noninvariance for the PHQ‐8 (e.g., lesbian, gay, bisexual, transgender, or queer identities). Moreover, the dichotomization of identities in this study may not be the most robust. Some of the variables may have had many more categories (e.g., education), and it is not clear that the groups here include all groups across which meaningful differences in the measurement of depression may exist.

**FIGURE A2:**
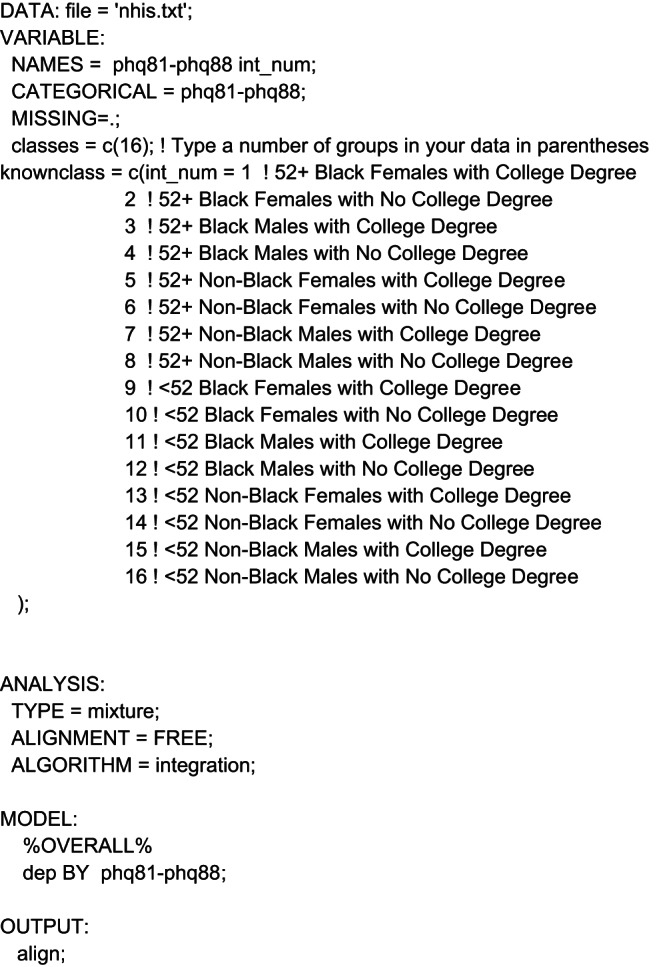
Mplus code for alignment method measurement invariance testing with categorical items.

Another limitation is that we treated items as continuous rather than categorical or ordinal. This choice is not without limitation, and the challenges of modeling ordinal data as continuous increase with Likert‐scaled items with fewer than 5 options.^41^ We treated these items as continuous to simplify the presentation of results, as our purpose was to illustrate the use of the alignment method. We do provide the code for implementing the alignment method where items are categorical in Figure A2 as well as the results in Table A2. We found that treating the items as categorical did not substantively change the interpretation of the main findings of this study. That is, using the 25% benchmark, the categorical treatment of items still implied trustworthy alignment results for the PHQ‐8, indicating that group‐specific factor means, and variances are comparable. When treating the items as categorical, there was less evidence of noninvariance and the invariance appeared more in the thresholds than loadings (i.e., roughly 9% for the thresholds and 2% for the loadings).

Although the alignment method provides a potential advancement in measurement invariance evaluation, the method comes with a new set of assumptions to understand and evaluate, and further methodological research on its efficacy and use is necessary.^8^, ^32^ For instance, as Flake and McCoach^32^ note, beyond the 25% benchmark, there is not much guidance for applied researchers to ascertain what constitutes approximate invariance or large noninvariance between groups. The 25% rule of thumb and *R* ^2^ effect sizes measures are based on limited empirical evidence and not yet well understood. Evaluators should use caution when comparing factor means and variances across intersectional subgroups if more than 25% of the items are noninvariant. Moreover, as previously mentioned, it is important to note that the intended use of the alignment method is to evaluate whether factor means and variance comparisons are meaningful across groups. As such, if a researcher is interested in understanding whether a particular item is non‐invariant in some way, then the alignment approach may not be the most appropriate method, and a differential item functioning analysis may be more suitable.^18^

In addition, extant research on the traditional measurement invariance approach suggests a minimum of 400 participants per group is required.^8^, ^28^, ^42^, ^43^, ^44^ In this study, we had group sizes as small as 209 and as large as 4195. However, it is not clear how many participants per group are needed for the alignment method or for intersecting identities. Additional research is essential to understand the appropriate sample sizes necessary as multiple comparisons increase both from larger numbers of items and groups. Last, even though the alignment method optimization procedures reduce the burden of performing measurement invariance evaluation, this functionality also creates the possibility of misuse and misinterpretation,^8^ and researchers should take care to check the assumptions of their analyses.

## 7 CONCLUSION

This study aimed to contribute to valid measurement in culturally diverse populations by demonstrating a method for intersectional measurement invariance testing and demonstrating its use on the PHQ‐8 where population subgroups were defined at the intersections of age, race, gender, and education. Using intersectionality as a guiding methodological framework, we investigated how a person's multiple identities and social positions might contribute to measurement noninvariance for the PHQ‐8. The alignment method was feasible for assessing intersectional measurement invariance, and computing aligned factor scores, across many groups. The PHQ‐8 demonstrated evidence of approximate measurement invariance across culturally diverse groups. We offer example code and discussion of how one might implement and interpret an intersectional measurement invariance testing approach in practice in a setting with many intersectional subgroups.

## APPENDIX A

**TABLE A1:** Patient Health Questionnaire (PHQ) depression instrument items.

Item	Item text
	Over the last 2 weeks, how often have you been bothered by …
PHQ‐1	Little interest or pleasure in doing things?
PHQ‐2	Feeling down, depressed, or hopeless?
PHQ‐3	Trouble falling or staying asleep, or sleeping too much?
PHQ‐4	Feeling tired or having little energy?
PHQ‐5	Poor appetite or overeating?
PHQ‐6	Feeling bad about yourself, or that you are a failure, or have let yourself or your family down?
PHQ‐7	Trouble concentrating on things, such as reading the newspaper or watching television?
PHQ‐8	Moving or speaking so slowly that other people could have noticed? Or the opposite, being so fidgety or restless that you have been moving around a lot more than usual?

*Note*: The response options for each item were not at all (1), several days (2), more than half the days (3), or nearly every day (4).

**TABLE A2:** Invariance results for aligned item threshold and loading parameters for PHQ‐1 to PHQ‐8 where items are treated as categorical.

Item thresholds			
Item (threshold)	Group invariance (non‐invariance)	# Non‐invariant groups	Invariance index
PHQ‐1 (1)	1 2 3 4 5 6 **(7)** 8 9 10 11 12 13 14 15 16	1	0.639
PHQ‐1 (2)	1 2 3 4 5 6 7 8 9 10 11 12 13 14 15 16	0	0.458
PHQ‐1 (3)	1 2 3 4 **(5)** 6 7 8 9 10 11 12 13 14 15 16	1	0.467
PHQ‐2 (1)	1 2 3 4 **(5)** 6 7 8 9 10 11 12 **(13)** 14 15 16	2	0.725
PHQ‐2 (2)	1 2 3 4 5 6 7 8 9 10 11 12 13 14 15 16	0	0.597
PHQ‐2 (3)	1 2 3 4 5 6 7 8 9 10 11 12 13 14 15 16	0	0.434
PHQ‐3 (1)	1 2 3 4 **(5)** 6 **(7)** 8 9 10 11 12 **(13)** 14 **(15)** 16	4	0.000
PHQ‐3 (2)	1 2 3 4 5 6 7 8 9 10 11 12 **(13)** 14 **(15)** 16	2	0.199
PHQ‐3 (3)	1 2 3 4 5 6 7 8 9 10 11 12 **(13) (14) (15) (16)**	4	0.190
PHQ‐4 (1)	**(1)** 2 3 4 **(5) (6) (7)** 8 9 10 11 12 **(13)** 14 15 16	5	0.446
PHQ‐4 (2)	1 2 3 4 **(5)** 6 7 8 9 10 11 12 13 14 15 16	1	0.406
PHQ‐4 (3)	1 2 3 4 5 6 7 8 9 10 11 12 13 14 15 16	0	0.535
PHQ‐5 (1)	1 2 3 4 5 6 7 8 9 10 11 12 13 **(14) (15) (16)**	3	0.591
PHQ‐5 (2)	1 2 3 4 5 6 7 8 9 10 11 12 13 14 15 16	0	0.632
PHQ‐5 (3)	1 2 3 4 5 6 7 8 9 10 11 12 13 14 15 16	0	0.468
PHQ‐6 (1)	1 2 3 4 **(5)** 6 7 8 9 10 11 12 13 **(14)** 15 **(16)**	3	0.196
PHQ‐6 (2)	1 2 3 4 **(5)** 6 7 8 9 10 11 12 13 14 15 16	1	0.539
PHQ‐6 (3)	1 2 3 4 5 6 7 8 9 10 11 12 13 14 15 16	0	0.396
PHQ‐7 (1)	1 2 3 4 **(5) (6) (7) (8)** 9 10 11 12 13 14 15 16	4	0.125
PHQ‐7 (2)	1 2 3 4 5 **(6)** 7 8 9 10 11 12 13 14 15 16	1	0.434
PHQ‐7 (3)	1 2 3 4 5 **(6)** 7 8 9 10 11 12 13 14 15 16	1	0.261
PHQ‐8 (1)	1 2 3 4 5 6 7 8 9 10 11 12 13 14 15 16	0	0.725
PHQ‐8 (2)	1 2 3 4 5 6 7 8 9 10 11 12 13 14 15 16	0	0.799
PHQ‐8 (3)	1 2 3 4 5 6 7 8 9 10 11 12 13 14 15 16	0	0.619

Factor loadings			
Item	Group (non)‐invariance	# Non‐invariant groups	Invariance index
PHQ‐1	1 2 3 4 5 6 7 8 9 10 11 12 13 14 15 16	0	0.085
PHQ‐2	1 2 3 4 5 6 7 8 9 10 11 12 13 14 15 16	0	0.283
PHQ‐3	1 2 3 4 5 6 7 8 9 10 11 12 (13) (14) (15) 16	3	0.255
PHQ‐4	1 2 3 4 5 6 7 8 9 10 11 12 13 14 15 16	0	0.000
PHQ‐5	1 2 3 4 5 6 7 8 9 10 11 12 13 14 15 16	0	0.155
PHQ‐6	1 2 3 4 5 6 7 8 9 10 11 12 13 14 15 16	0	0.021
PHQ‐7	1 2 3 4 5 6 7 8 9 10 11 12 13 14 15 16	0	0.000
PHQ‐8	1 2 3 4 5 6 7 8 9 10 11 12 13 14 15 16	0	0.127

*Note*: The group values correspond to the intersectional coding. The bolded numbers in parentheses refer to the groups that show significant non‐invariance for the parameter. The invariance index is *R* ^2^. An *R* ^2^. close to 1 provides evidence that there is complete invariance. Conversely, an *R* ^*2*^. near 0 provides evidence that group mean differences explain little to no variability in item parameters.
